# The effect of data complexity on classifier performance

**DOI:** 10.1007/s10664-024-10554-5

**Published:** 2024-10-31

**Authors:** Jonas Eberlein, Daniel Rodriguez, Rachel Harrison

**Affiliations:** 1 School of Technology, Oxford Brookes University, Headington Campus, Oxford, OX3 0BP UK; 2 Dept of Computer Science, University of Alcala, Alcalá de Henares, Madrid, 28805 Spain

**Keywords:** Classification, Software defect prediction, Data complexity metrics

## Abstract

The research area of Software Defect Prediction (SDP) is both extensive and popular, and is often treated as a classification problem. Improvements in classification, pre-processing and tuning techniques, (together with many factors which can influence model performance) have encouraged this trend. However, no matter the effort in these areas, it seems that there is a ceiling in the performance of the classification models used in SDP. In this paper, the issue of classifier performance is analysed from the perspective of data complexity. Specifically, data complexity metrics are calculated using the Unified Bug Dataset, a collection of well-known SDP datasets, and then checked for correlation with the defect prediction performance of machine learning classifiers (in particular, the classifiers C5.0, Naive Bayes, Artificial Neural Networks, Random Forests, and Support Vector Machines). In this work, different domains of competence and incompetence are identified for the classifiers. Similarities and differences between the classifiers and the performance metrics are found and the Unified Bug Dataset is analysed from the perspective of data complexity. We found that certain classifiers work best in certain situations and that all data complexity metrics can be problematic, although certain classifiers did excel in some situations.

## Introduction

The field of Software Defect Prediction (SDP) offers automated solutions to identify defects or issues in software, and has grown in popularity alongside the importance of software itself (Chen et al. 2018). The value in terms of cost and resource savings and improvement of systems has been recognised in literature and business (Hall et al. 2011; Hosseini et al. 2017). Moreover, it has even been found that the effectiveness of automated defect identification is significantly better than when detection is under manual control (Menzies et al. 2007; Misirli et al. 2011).

However, no matter the effort in these areas, it seems that there is a ceiling in the performance of classification models used in SDP, and various directions are being explored in order to find performance improvements (Menzies et al. 2010). It has been established that the performance of models is highly data dependent and that each model has different strengths and weaknesses based on the characteristics of the underlying data (Cavalcanti et al. 2012). This has also been discussed by Wan et al. (2023).

This is also related to the concept of meta-learning, where knowing about the characteristics of the data may help to select different models or apply different pre-processing techniques to boost the performance of the classifiers. In the field of SDP, software metrics can be used to build models and predict defects. For this, a number of different measures exist. However, in addition to metrics collected from the source code or configuration management systems, the so called *data complexity metrics* can be calculated to further characterise data to give an indication of data complexity. Here, we use the term *data complexity* to refer to the part of software complexity concerned with the data which is in use, as measured by *Balance, Dimensionality, Linearity, Neighborhood, Network* and *Overlap* (see § 3.2). Thus, data complexity indicates a more challenging situation for classification models. These measures have been developed and expanded and applied in different scenarios (Ho and Basu 2002).

This is partially related to the concept of data quality where a number of data issues such as imbalance, overlap, dimensionality or separability have been addressed in the literature of SDP separately, usually applying different pre-processing techniques as a way to improve the performance of the classifiers. There are other issues such as missing values, concept drift, duplicates instances, or outliers present in SDP datasets also addressed in the literature that are not directly measured with the data complexity metrics used in this work. Bhandari et al. (2022) address data quality issues in a systematic literature review in the context of SDP.

Within this paper, data complexity metrics (from the metalearning point of view) are correlated with the performance of Machine Learning (ML) classifiers. Hence, data complexity environments are identified which allow better or worse performance for each classifier. The paper focuses on the complexity of the software datasets used for SDP, particularly the specific characteristics that exist in the data which can create different challenges. In the literature, various research has been conducted into measures for datasets, as well as pre-processing and the competence of classifiers. Further, research has been performed on measures for datasets to try to identify classifier performance (Ali and Smith 2006; Luengo and Herrera 2015). This paper suggests a method that can help to achieve improved classification results and also helps with the understanding of classifiers. Seminal work was published by Ho and Basu (2002), on the topic of complexity metrics. Further, Luengo and Herrera (2015) used these metrics to predict the behaviour of classifiers. Lorena et al. (2019) then extended the original complexity metrics.

This work contributes to the literature by assessing the relationship between data characteristics and classifier performance specifically for the research area of SDP. Further, little research has been performed using the refined complexity metrics of Lorena et al. (2019), which we use and which have been referred to as state-of-the-art (Pascual-Triana et al. 2021). We also identify the domains of competence for the classifiers.

The following research questions were developed in the context of SDP.

- RQ1: Does data complexity influence classifier performance?
- RQ2: What are the domains of competence for the classifiers?

To answer these research questions, this paper is structured as follows. Next, a background on the topic, including a literature review, is provided in Section 2. Thereafter, the methodology is discussed and specified in Section 3. Then, in Section 4 the results are shown and discussed together with seminal work in literature. Finally, conclusions, limitations and suggestions for future work are discussed in Section 6.

## Background

In SDP, the use of different classifiers and their optimisation has been the main concern for the optimisation of a model. Throughout the literature, all kinds of classifiers have been applied and tested in combination with different datasets (Ali and Smith 2006). From this and the machine learning literature, it can be concluded that no single classifier can be considered universally applicable and optimum in all cases (Ho and Basu 2002; Ali and Smith 2006). Rather, certain classifiers work best in certain situations (Ho and Basu 2002). Luengo and Herrera (2010) refer to this as the *domain of competence* of a classifier and this is connected to the *no free lunch theorem* (Wolpert and Macready 1995).

As well as the characteristics and applicability of the classifiers themselves, the performance assessment of the classifiers (their *goodness*) is another important factor in the optimisation of a model (Rodriguez et al. 2014). Arisholm et al. (2010) found that the use of different *evaluation measures* or *performance measures* can lead to different results and conclusions about classifiers.

SDP can be approached as as a classification problem, i.e., a binary problem (defect or no defect) or as a regression problem with a continuous dependent variable (the number of defects). The following section further explores this specific area of interest in more detail.

### Software Defect Prediction

The AI-based techniques for SDP are usually based on ML algorithms or statistical models (Misirli et al. 2011). Using such, various combinations of classifiers and datasets have been explored in the literature in an effort to find which classifiers may be better suited for datasets with certain characteristics. However, Menzies et al. (2010) identify a ceiling that has been reached in terms of performance of the classifiers and many authors have concluded that no single best classifier exists (Ali and Smith 2006; Luengo and Herrera 2015). Nonetheless, authors have found that defect prediction tools can identify up to 70 percent of the defects in software (Menzies et al. 2007), while manual inspection and review was found to identify significantly fewer (Fagan 1976; Shull et al. 2002). Accordingly, defect prediction systems are seen as valuable to Quality Assurance (QA) in particular and the software development process in general (Menzies et al. 2010).

To capitalise on SDP, (Ma et al. 2018) suggest a structured SDP procedure that is made up of three stages: (1) extraction of the data set, (2) creation of a detection model, and (3) detection and evaluation of defects.

For SDP, numerous datasets are used in the literature. Such data is historical, and so can be used to train a classifier (Ma et al. 2018). Recently, Ferenc et al. (2020) have done a unification of various publicly available datasets in order to compare them and to assess the cross-project applicability of classifiers and data sets. Song et al. (2019) specifically focus on the imbalanced nature of SDP data sets, pointing out that they are imbalanced due to low numbers of defects. This *imbalance problem* comes with numerous challenges when applying classifiers and ML algorithms. Such challenges may be tackled by pre-processing the data (balancing or cost dependant techniques), classifiers which are robust to the imbalance problem and measuring the ’goodness’ of the classifiers with different evaluation metrics. The imbalance problem is a challenge, as data mining algorithms work under the assumption of balanced data sets. This also can be a domain of competence of a classifier, and hence may be an area in which the classifier excels.

Britto et al. (2014) present a literature review of classifier systems, organized according to a new taxonomy. The authors propose the use of a dynamic selection approach (selecting specific classifiers for each test) and investigate whether a relationship exists between the performance and the complexity of the classification problem. Their results suggest that a relationship between the observed performance contribution and the complexity of the classification problem does exist.

Despite the aforementioned benefits and positive aspects of SDP, Hosseini et al. (2017) found that many companies are unable to consider SDP systems as they do not have access to the data and the preparation of data is resource intensive. As an alternative, data can be used from other projects (this is referred to as *cross-project* SDP). Turhan et al. (2013) and Menzies et al. (2010) even find that cross-project SDP can perform better than projects using within-project data. Chen et al. (2018) also suggest that this may be a solution to the imbalance problem and find in the literature that cross-project SDP has a higher defect-detection rate, possibly because the cross-project data provides more detailed information on the defective class. Using datasets from other projects does have the effect of reducing the resource investment, however it does come with challenges (Hosseini et al. 2017).

### Data Complexity Metrics

Throughout the literature, it is accepted that classification problems are highly data dependent. In line with this, complexity metrics were introduced to assess the characteristics of datasets. Prior to this, statistical tools were used to describe data, but this was seen to be unnecessarily complicated (Cavalcanti et al. 2012). According to Ma et al. (2018), the concept of data complexity metrics is derived from data mining. Hence, it is a concept that is rarely applied within the SDP literature. However, data complexity metrics are commonly used for prediction problems (Fenton and Neil 1999; Misirli et al. 2011) and Ho and Basu (2002) report that the characteristics of the data determine the difficulty of such problems. Hence, the performance and behaviour of classifiers correlates with and depends on the complexity of the data (Sotoca et al. 2005). We explore this issue within the context of SDP.

The paper by Ho and Basu presents 12 complexity metrics that can be used to assess data. With the use of these complexity metrics, the authors hope to attain a holistic view of the problem difficulty that is being faced (Luengo and Herrera 2015). Specifically, the *multifaceted nature* of problems is identified. Another paper by Ho et al. (2006) further underlines the importance of using a variety of measures. Due to the complexity metrics being independent from the base classifier, it is possible to use the complexity metrics to identify a suitable base classifier. Furthermore, a method for deciding a classifier based on the complexity metrics is also suggested by Luengo and Herrera (2010). In addition, the same authors suggest that complexity metrics can be used to establish *domains of competence* of classifiers, and hence the situations and environments in which they perform well. In the work of Ma et al. (2018) with respect to SDP, complexity metrics can be used to describe the *difficulty of detection*. As an extension of this, the complexity metrics can be utilised to identify the need for pre-processing steps or other changes to the data (Pascual-Triana et al. 2021). Furthermore, they can help to understand the quality of the results attained in classification (Pascual-Triana et al. 2021) and also indicate the reliability of the performance.

Ho and Basu categorise the original set of measures into three groups: (1) measures of the overlap of individual feature values, (2) measures of the separability of classes and (3) the geometry, topology and density of manifolds. A similar categorisation is described by Luengo and Herrera (2010). Ho et al. (2006) also define three broader sources of difficulty, these being (1) class ambiguity (classes cannot be distinguished between using the given features), (2) boundary complexity (Kolmogorov complexity, geometrical descriptors), and (3) sample sparcity and feature space dimensionality (lack of samples, especially in high dimensionality spaces).

Further measures are added and classified into measures for feature-correlation, linearity, neighborhood, network, dimensionality, balance and overlap in Lorena et al. (2018). Pascual-Triana et al. (2021) refer to this last set of complexity metrics as the state-of-the-art metrics, emphasizing their relevancy. These metrics are widely used in the field (Ho and Basu 2002; Sotoca et al. 2005; Garcia et al. 2018). According to Cavalcanti et al. (2012), it is necessary to have multiple and different measures, as no single measure is able to explain the complexity of data completely. Nonetheless the focus in the literature remains on balance measures and overlap measures. Consequently we calculated metrics on balance, dimensionality, linearity, neighborhood, network and overlap related to classification complexity. Details of the metrics are given in the next section.

The recent paper by Wan et al. (2023) defines data complexity to include both instance hardness (correct classification of instances) (Arruda et al. 2020) and dataset complexity (overall difficulty of learning a concept from a dataset) (Smith et al. 2014). Wan et al. suggest that:

- disparities across datasets require consideration,
- class overlap is the primary factor contributing to instance hardness,
- feature and structural overlap are primary factors contributing to data complexity and
- there is no single data pre-processing method that can be recommended in all cases.

In fact, the paper also suggests that data pre-processing may not always reduce data complexity. In contrast, Agrawal and Menzies (2018) suggest that data pre-processing can be more important than classifier choice.

### Machine Learning Classifiers

The classifiers selected in this work are all of different types. This was an intentional decision to clearly differentiate between the domains of competence. Aligning with Kam Ho and Bernadó-Mansilla (2006), the classifiers used are not an exhaustive list. Rather, this is a point from which exploration can be made. These classifiers are commonly used in the literature and other researchers have also used them (e.g., (Hammad 2021; Challagulla et al. 2008)).

A popular base classifier for research with imbalanced data sets is the decision tree classifier C5.0. This is an improved version of the C4.5 and ID3 classifiers by Quinlan (1993). In our work the leaves of the tree correspond to classes, and the nodes correspond to attributes forming branches. Attribute selection is embedded into the classifier using the gain ratio as a criterion at every node of the tree. In addition to the seminal works by Quilan, it is thoroughly explained in Ali and Smith (2006).

The Naive Bayes (NB) classifier is a probabilistic classifier which uses Bayes theorem to predict the class for each case. A Naive Bayes classifier assigns a set of attributes $A_1,\dots ,A_n$ to a class *C* such that $P(C | A_1,\dots ,A_n)$ is maximum. It is extensively used in the literature and was found to perform well with imbalance by Menzies et al. (2007) and Rodriguez et al. (2014). Naive Bayes is well explained, for example, in Webb et al. (2010). Menzies, Menzies et al. (2007) and Rodriguez et al. (2014) find that the Naive Bayes classifier shows good performance. However, it is reported that the performance of this base classifier could be improved with pre-processing.

Japkowicz and Stephen (2002) find that the Artificial Neural Networks (ANN), Decision Tree (C5.0), and Support Vectors Machines (SVM) classifiers are vulnerable to imbalanced datasets. Support Vector Machines (SVM) can be regarded as hyperplanes in a high-dimensional feature space (Shawe-Taylor and Cristianini 2002). Support Vector Machines are extensively used in the literature.

Ali and Smith (2006) show that the ANN classifier performs best across different performance measures, and that the Decision Tree classifier (C5.0) and SVM classifier also performed well. However, they could not find statistically significant differences between classifiers. As part of their research, they propose a “rule-based classifier selection approach" (Ali and Smith 2006). In a balanced environment, Luengo and Herrera (2015) apply the Decision Tree classifier C5.0, as well as the SVM and *k*-Nearest Neighbor classifiers. Fernández-Delgado et al. (2014) also report that there is no need for a large number of classifiers.

The Random Forest (RF) classifier is a classifier consisting of a collection of tree-structured classifiers (Breiman 2001). Each tree casts a single vote for the most popular class at a specific input. Again it is extensively used in the literature. Khoshgoftaar et al. strongly recommend the use of random forest classifiers when learning from imbalanced data (Khoshgoftaar et al. 2007).

The five classifiers C5.0, NB, ANN, RF and SVM were chosen for this research because they have become de-facto industry standard classifiers in machine learning, they are also of different type.

### Performance Metrics

The F-measure and Matthew’s Correlation Coefficient (MCC) are relevant performance metrics, and were used in this work as they are widely adopted and have been found to be relevant for SDP (Lavazza and Morasca 2022). Lavazza and Morasca (2022) also conclude that MCC is a sensible and useful metric for assessing the performance of binary classifiers. The choice of metrics is impacted by the fact that most SDP-datasets are imbalanced (Ali and Smith 2006; Rodriguez et al. 2014; Mahmood et al. 2015). The F-measure, however, has also been criticized as it completely ignores the true negatives (Christen et al. 2023).

## Methodology

In this section we describe our method, the datasets we used, the data complexity metrics, the machine learning classifiers used as well as our experiment and performance metrics. We want to know how certain classifiers work in certain situations with the chosen data complexity metrics. We also want to investigate any inherent imbalance in the chosen SDP datasets.

**Fig. 1:**
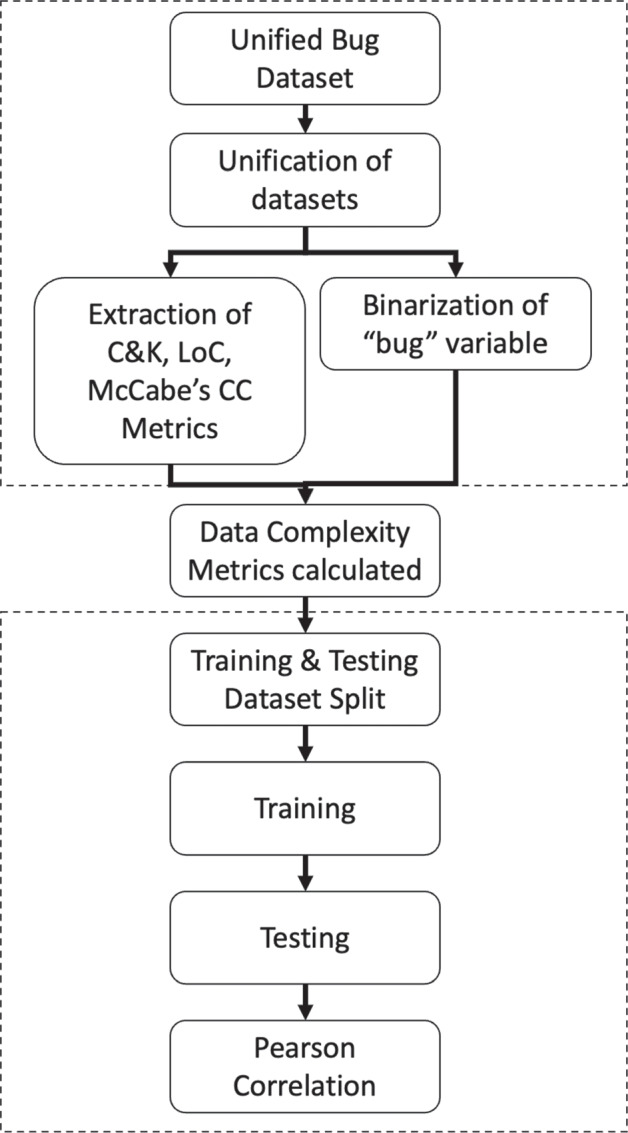
Flowchart of methodological approach

**Table 1:** Selected Datasets from UBD

*Dataset Group*	*Dataset*	*# Instances*	*Imb. Ratio*
BugPrediction	Eclipse	997	0.21
BugPrediction	Equinox	319	0.39
BugPrediction	lucene	846	0.28
BugPrediction	Mylyn	1405	0.15
BugPrediction	PDE	1491	0.14
Github	Android	73	0.27
Github	antlr4	479	0.04
Github	broadleaf	1593	0.18
Github	ceylon	1610	0.04
Github	elasticsearch	5908	0.11
Github	hazelcast	3412	0.11
Github	Junit	731	0.05
Github	mapdb	331	0.12
Github	mcMMO	301	0.19
Github	Neo4j	5899	0.01
Github	Netty	1143	0.24
Github	orientdb	1847	0.15
Github	oryx	533	0.14
Github	titan	1468	0.07
PROMISE	ant14	178	0.22
PROMISE	camel16	1058	0.21
PROMISE	ivy20	352	0.11
PROMISE	jedit43	492	0.02
PROMISE	log4k12	205	0.92
PROMISE	lucene24	1011	0.26
PROMISE	pbeans2	51	0.20
PROMISE	poi30	442	0.64
PROMISE	synapse12	276	0.33
PROMISE	velocity16	260	0.38
PROMISE	xerces20	546	0.73

### Dataset

The Unified Bug Dataset curated by Ferenc et al. (2020) was chosen as the dataset for this work. The Unified Bug Dataset is composed of a collection of well-known public datasets in SDP. The data set consists of numerous source code metrics that were obtained through source code analysis (Ferenc et al. 2020).

As these datasets are derived from different backgrounds, their software metrics and information about them differ (Ferenc et al. 2020). The unification in the Unified Bug Dataset was done by standardising the software metrics that are provided (Fig. 1). For this paper, the C&K metrics were chosen as the software metrics that should be extracted in the early stage (Chidamber and Kemerer 1994). These software metrics are probably the most used ones in the SDP literature. Later, Lines of Code (LoC) and McCabe’s CC were extracted in addition. Finally and importantly, the metric *bug* (the class attribute) was extracted, indicating whether a bug exists in a software class. As this variable was recorded as continuous, it was converted to a binary variable. Specifically, if no bug existed, the value 0 was recorded whereas if one or more bugs existed, the value was replaced by 1. All of the metrics used here were part of the actual Unified Bug Dataset (Ferenc et al. 2020) and the actual datasets used in this work are listed in Table 1. The final column (*Imb. Ratio*) shows the extent to which the data set is unbalanced.

### Data Complexity Metrics: Details

- **Balance:** this refers to the distribution of samples among the classes. Two metrics are utilised To measure balance (Lorena et al. 2018, 2012):
  1. **‘Entropy of Class Proportions’ (C1)** is an estimate of the normalised entropy of the class size distribution. Specifically, the value 0 indicates perfect imbalance, so all samples belong to one class, whereas a value of 1 indicates perfect balance between the classes.
  2. **‘Imbalance Ratio’ (C2)** as defined by Lorena et al. (2018) measures imbalance, with high values indicating high imbalance.
  For both metrics, high imbalance is associated with high complexity.
- **Dimensionality:** these metrics describe the sparsity of the data (“sparse datasets tend to have regions of low density" (Lorena et al. 2012)). These regions are challenging to classifiers and hence are relevant as complexity metrics. For all three measures, a high value indicates a more complex problem (Lorena et al. 2018).
  1. **‘Average Number of Points per Dimension’ (T2)** is the ratio between the dimensionality of the examples and the number of examples.
  2. **‘Average Number of Points per PCA’ (T3)** is as above except that the number of PCA components representing 95 percent variability is used as the numerator.
  3. **‘Ratio of the PCA Dimension to the Original’ (T4)**, is the ratio between the relevant dimensions chosen in PCA and the original dimensions.
- **Linearity:** these metrics show how easy it is to separate different classes. Linearity metrics are the only metrics that provide classification rather than regression. Linearly separable problems are considered to be less complex. For all metrics, large values indicate high complexity (Lorena et al. 2018).
  1. **‘Sum of the Error Distance by Linear Programming’ (L1)** is the distance between the class and the linear boundary.
  2. **‘Error rate of the linear classifier’ (L2)**, uses the linear classifier SVM.
  3. **‘Non-linearity of a linear classifier’ (L3)** creates a new dataset randomly interpolating pairs of training examples of the same class and then induces a linear SVM on the original data and measures the error rate in the new data points.
- **Neighborhood:** these metrics rely on distances between neighborhoods, overlaps and decision boundaries. In total, six measures were used (Lorena et al. 2018):
  1. **‘Fraction of Borderline Points’ (N1)** computes the percentage of vertices incident to edges connecting examples of opposite classes in a Minimum Spanning Tree (MST). A high value indicates more complexity.
  2. **‘Ratio of Intra/Extra Class Nearest Neighbor Distance’ (N2)** is the ratio of the distance of each instance and its nearest neighbor to the distance of each instance and its furthest neighbor. A high value indicates a complex problem.
  3. **‘Error Rate of the Nearest Neighbor’ (N3)** first applies a 1NN algorithm and then extracts its error rate. A high value indicates greater complexity.
  4. **’Non-linearity of the Nearest Neighbor Classifier’ (N4)** uses the same method as L3, but uses a 1-NN classifier. The interpretation also is the same.
  5. **‘Fraction of Hyperspheres Covering Data’ (T1)**, builds and counts hyperspheres and then compares this to the number of examples. Large values are associated with high complexity.
  6. **‘Local Set average Cardinality’ (LSC)** is the average of the set of points whose distance is smaller than the distance of the other class. Here, high scores indicate simpler problems.
- **Network:** the epsilon-NN algorithm is used to transform the data to a graph (Morais and Prati 2013). This is a popular approach as many real world problems can be depicted this way  (Lorena et al. 2018).
  1. **‘Average Density of the Network’ (Density)** is the number of edges in the graph, divided by the maximum number of edges between pairs of points. High values indicate high complexity.
  2. **‘Clustering Coefficient’ (ClsCoef)** averages the clustering tendency of the vertices by the ratio of edges between its neighbors and the total number of edges that could exist. Again high values indicate high complexity.
  3. **‘Hubs Score’ (Hubs)** measures the connectedness of the vertices. The more vertices a vertex is connected to the higher the score. If these vertices are also highly connected, the score is increased even more.
- **Overlap** refers to the usefulness of the information given in separating classes. This indicates the discriminating power of the features (Lorena et al. 2018). A high number of disriminative features simplifies a dataset. Five measures are used (Lorena et al. 2018):
  1. **‘Maximum Fisher’s Discriminant Ratio’ (F1)** measures the overlap between the features and takes the value of the largest ratio. High values indicate high complexity.
  2. **‘Directional-vector maximum Fisher’s discriminant ratio’ (F1.v)** is a version of F1 that searches for a separating vector. Values can range from 0 to 1, with high values indicating high complexity. High complexity means that the separation of data is more difficult and unclear (Lorena et al. 2018).
  3. **‘Volume of the overlapping region’ (F2)** is identified by finding the minimum and maximum values for each feature. Again a high value indicates high complexity.
  4. The **‘maximum individual feature efficiency’ (F3)** selects the most discriminative feature in the data. High values of this measure indicate high complexity.
  5. **‘Collective feature efficiency’ (F4)** is the ratio of examples that have been found divided by the maximum individual feature efficiency. Again high values indicate high complexity.

### Experimental Work

A stratified hold out approach for the experimental work was followed, i.e., the splitting of datasets into training and testing sets was implemented and balancing was performed for the bug attribute as the variable of interest. Although a more complex approach could have been followed such as cross validation, the focus of this work is analysing the complexity of the software defect datasets rather than getting the most accurate classification.

The results for additional performance metrics can be found in the replication package.

### Statistical Correlation

Pearson’s Correlation coefficient was run to study the correlation of the complexity metrics and the performance metric outcomes of the classifiers. Pearson’s Correlation was chosen following the work of Pascual-Triana et al. (2021) who also applied this method in their research to study the correlation of complexity metrics with classifier performance.

## Results and Discussion

The following section shows the summary statistics of the complexity metrics and the classifier performance with the datasets. For each, the minimum, maximum, mean and standard deviation are provided and discussed. For the complexity metrics, we focus on the results that values have in terms of data complexity.

**Table 2:** Summary Statistics for Balance Measures

	*Min*	*Max*	*Mean*	*S.D.*
C1	0.04	0.97	0.59	0.28
C2	0.08	0.99	0.60	0.27

**Table 3:** Summary Statistics for Dimensionality Measures

	*Min*	*Max*	*Mean*	*S.D.*
T2	0.00	0.18	0.02	0.04
T3	0.00	0.02	0.00	0.00
T4	0.11	0.22	0.12	0.03

### Summary Statistics for Complexity Metrics

As it can be observed in Table 2 for the balance measure *entropy of class proportions* (C1), large deviations exist in terms of the balance of the datasets. The mean is relatively central between perfect imbalance (0) and perfect balance (1). Further, the minimum and maximum show that datasets with both extremes exist. As a higher imbalance is synonymous with complexity in this topic area, no clear conclusion can be drawn from these measures. For the other balance measure (the imbalance ratio C2), the larger values signal imbalance and thereby higher complexity. An average (mean) of 0.60 indicates that the datasets are in between being balanced and imbalanced. The standard deviation, as well as minimum and maximum values, shows that both extremes do exist.

Table 3 shows the summary for the dimensionality factor. For the first measure of dimensionality, T2, negative values can be attained when the number of features is smaller than the number of examples, and the maximum value is the number of examples the dataset has to offer. Here, higher values indicate more complex problems. T3 is also dependent on the number of examples, as it uses the number of PCA components to achieve 95% variability. For T4, larger values indicate that more original features are needed for the description of variability in the data. This is synonymous with complexity. The range of T4 is 0 to 1, and with a mean of 0.12. Low values exist in these datasets and indicate that the problems are quite simple with regards to data sparsity (Lorena et al. 2018).

**Table 4:** Summary Statistics for Linearity Measures

	*Min*	*Max*	*Mean*	*S.D.*
L1	0.01	0.39	0.22	0.16
L2	0.00	0.19	0.10	0.06
L3	0.00	0.18	0.10	0.05

For all the complexity measures focusing on linearity (see Table 4), higher values indicate higher complexity. As the values are low (L1 = 0.2175; L2 = 0.1049; L3 = 0.0981), low complexity in regards to linearity was found in the datasets that are part of the Unified Bug Dataset.

**Table 5:** Summary Statistics for Neighborhood Measures

	*Min*	*Max*	*Mean*	*S.D.*
N1	0.01	0.50	0.26	0.15
N2	0.08	0.41	0.28	0.09
N3	0.01	0.31	0.16	0.09
N4	0.00	0.30	0.12	0.07
T1	0.00	0.04	0.01	0.01
LSC	0.69	1.00	0.94	0.07

In the case of the six measures of the neighborhood (Table 5), the interpretation of the values differs. The value attained for ’fraction of borderline points’ (N1) is 0.26, which indicates that simpler decision frontiers are sufficient. Higher values for N2 indicate higher complexity. With a mean of 0.2792, the datasets are rather simple according to this measure. According to Okimoto et al. (2017), a high error rate of the nearest neighbor (N3) indicates that many examples are close to examples of other classes, making the problem more complex. However, the mean error rate of 0.1623 is rather low. Next, the higher the value for the N4 algorithm, the higher the complexity. In the case of this research, the value is 0.1178, indicates low complexity. As above, a larger ’fraction of hyperspheres covering data’ (T1) is associated with higher complexity. The value (0.0097) is very low, indicating simplicity. Finally for neighborhood, a lower ’Local Set Average Cardinality’ indicates higher complexity. As a high score (0.9449) was found, simplicity is indicated. Accordingly, the relevant datasets are simple in terms of all complexity measures used for neighborhood.

Table 6 shows the result for the network complexity category. The first network complexity measure is Density, where large values indicate higher complexity. With a mean of 0.8380, the datasets can be categorised as rather complex in this regard. The mean Clustering Coefficient (ClsCoef) on the other hand is rather low (0.3657), and the interpretation of values is the same as for the above measure. Finally, the high Hubs score of 0.9116 means that higher density exists, reducing complexity.

**Table 6:** Summary Statistics for Network Measures

	*Min*	*Max*	*Mean*	*S.D.*
Density	0.79	0.88	0.84	0.03
ClsCoef	0.29	0.45	0.37	0.04
Hubs	0.84	0.96	0.91	0.03

**Table 7:** Summary Statistics for Overlap Measures

	*Min*	*Max*	*Mean*	*S.D.*
F1	0.89	1.00	0.96	0.03
F1.v	0.04	0.64	0.39	0.14
F2	0.00	0.10	0.02	0.03
F3	0.25	1.00	0.85	0.19
F4	0.11	0.99	0.74	0.27

Five different measures are used for the overlap complexity (Table 7). The mean and the minimum value of Fisher’s discriminant ratio (F1) are high, indicating that at least one of the attributes enables the learner to separate the different classes (Orriols-Puig et al. 2010). The rather high values found here indicate a complex problem. For F1.v, a variant of F1 which uses a "Directional Vector", lower values compared to F1 were found (mean=0.39), indicating less complexity. This can be explained by the different approach of the measure. As discussed before, F2 is the volume of the overlapping regions. With a mean of 0.0173 and a maximum of 0.1, low values are present throughout the datasets. Low values signal low overlap and less complexity (Okimoto et al. 2017). For the ’maximum individual feature efficiency’ (F3) a mean of 0.8468 was found, meaning the problem can be categorised as complex. However, the standard deviation is high. The same interpretation for complexity and standard deviation can be made for the ’collective feature efficiency’ (F4) (Lorena et al. 2018).

To sum up, the summary statistics have provided some interesting insights into the complexity metrics and the datasets that are part of the Unified Bug Dataset. We found the mean imbalance level for these datasets to be lower than expected, as SDP datasets were thought to be highly imbalanced. Although highly imbalanced datasets exist in this research, so do balanced ones. This may inspire further exploration of the relationship between the complexity metrics and the classifiers’ performance. Regarding the other complexity metrics it was found that the datasets are rather simple on average in terms of dimensionality, linearity and neighborhood. On the other hand, the datasets are generally complex when looking at feature correlation and overlap measures. Contradictory results were found for some of the categories.

### Performance Summary Statistics for Classifiers

For all the classifiers, similar performance was found for the two evaluation measures used (see Table 8). Here, the interpretation of the results differs as the F-measure has a range from 0 to 1 and MCC a range from -1 to 1.

**Table 8:** Summary Performance statistics for all Classifiers

	*C5.0*		*NB*		*ANN*		*RF*		*SVM*	
	*f-measure*	*MCC*	*f-measure*	*MCC*	*f-measure*	*MCC*	*f-measure*	*MCC*	*f-measure*	*MCC*
*Min*	0.61	-0.05	0.53	-0.08	0.00	-0.17	0.22	-0.04	0.43	-0.03
*Max*	1.00	0.57	0.99	0.73	1.00	0.76	1.00	0.62	1.00	0.53
*Mean*	0.88	0.29	0.84	0.29	0.85	0.30	0.87	0.36	0.88	0.28

**Table 9:** Pearson Correlation Results for Complexity Metrics

	*C5.0*		*NB*		*ANN*		*RF*		*SVM*	
	*f-measure*	*MCC*	*f-measure*	*MCC*	*f-measure*	*MCC*	*f-measure*	*MCC*	*f-measure*	*MCC*
*Imbalance*										
C1	-.82$\star$	.40$\star$	-.63$\star$	.12	-.32$\bullet$	.45$\star$	-.35$\bullet$	.35$\bullet$	-.57$\star$	.41$\bullet$
IMC2	.86$\star$	-.37$\bullet$	.63$\star$	-.13	.34$\bullet$	-.43$\star$	.36$\star$	-.31	.64$\star$	-.39$\star$
*Dimensionality*										
T2	-.39$\star$	-.19	-.17	.14	-.25	.03	-.13	.26	-.06	.45$\star$
T3	-.39$\star$	-.20	-.16	.13	-.24	.02	-.13	.27	-.05	.43$\bullet$
T4	.12	-.18	.06	-.19	.11	-.12	.10	.05	.11	-.47$\star$
*Linearity*										
L1	-.83$\star$	.37$\bullet$	-.68$\star$	.09	-.34$\bullet$	.37$\bullet$	-.39$\star$	.26	-.62$\star$	.27
L2	-.81$\star$	.37$\bullet$	-.67$\star$	.07	-.34$\bullet$	.35	-.38$\star$	.24	-.60$\star$	.22
L3	-.80$\star$	.37$\bullet$	-.67$\star$	.06	-.33$\bullet$	.33	-.39$\star$	.21	-.60$\star$	.15
*Neighborhood*										
N1	-.76$\star$	.09	-.66$\star$	-.02	-.33$\bullet$	.16	-.36$\star$	.12	-.45$\star$	.30
N2	-.65$\star$	.05	-.70$\star$	-.03	-.46$\star$	-.15	-.48$\star$	-.06	-.38$\star$	.21
N3	-.74$\star$	.12	-.57$\star$	.07	-.37$\star$	.18	-.35$\bullet$	.25	-.45$\star$	.4$\bullet$
N4	-.65$\star$	.15	-.61$\star$	-.14	-.20	.05	-.27	.04	-.43$\star$	.08
T1	.02	-.28	.11	.22	-.12	-.10	-.02	.16	.07	.36
LSC	-.31$\bullet$	.41$\star$	-.34$\bullet$	-.13	-.17	.15	-.20	.17	-.11	.14
*Networking*										
Density	-.84$\star$	.25	-.64$\star$	.10	-.32$\bullet$	.40$\bullet$	-.34$\bullet$	.32	-.57$\star$	.38$\bullet$
ClsCoef	.08	-.34$\bullet$	-.06	.03	-.37$\star$	-.41$\bullet$	-.23	-.23	.43$\star$	.34
Hubs	-.07	.26	.10	.09	.12	.41$\bullet$	.12	.29	-.06	.33
*Overlap*										
F1	.28	-.02	-.03	-.38$\star$	.00	-.29	-.10	-.44$\star$	.05	-.68$\star$
F2	.13	.26	.04	.05	.10	-.08	.15	.20	.14	-.10
F3	-.07	.25	-.09	-.24	.10	.04	.05	-.13	-.01	-.40$\star$
F4	.05	.31	.01	-.26	.26	.11	.16	-.13	.03	-.47$\star$

### Discussion

#### Research Question 1

Table 9 combines the results for Pearson’s Correlation coefficients of all the complexity metrics. The results for each category of complexity metrics are discussed below.

To understand the influence of imbalance on classifier performance, the correlation of two complexity measures with the classifier performance is utilised. The first, the ’entropy of class proportions’ (C1) value, is negatively and significantly correlated with all of the F-measures, contrary to what is expected (the more imbalanced the data, the better the classifier performance; see  3.2). C1 is positively and significantly correlated with most of the MCC-measures, as would be expected from prior research (the more balanced the data, the better the classifier performance).

Secondly, for the imbalanced ratio (IMC2), a statistically significant positive correlation was found with the F-measures. As the interpretation of IMC2 opposes the one of of C1 (see § 3.2), here, more imbalanced datasets are associated with better classifier performance, matching the results from C1. Again however, opposing correlations were found with the MCC measures.

Throughout the literature the consensus is that imbalance negatively affects classifier performance, but this is only partially confirmed here, mostly by the MCC measures. For example, Lorena et al. (2012) find the balance complexity measures to be particularly affected, and report that their model performance is positively correlated when the data has greater balance. In Pascual-Triana et al. (2021), the mentioned negative effect of imbalance on the C5.0 and Naive Bayes classifiers is observed. Quinlan (1993) argues that the lack of performance of C5.0 with imbalanced data only occurs because C5.0 aims to optimise accuracy. The majority of researchers find imbalance to negatively affect classifier performance. In our research this negative effect is only statistically significant for the C1 MCC measures for C5.0, ANN, RF and SVM.

For the category ’dimensionality’, three measures were used, for all of which a higher value indicates higher complexity (see § 3.2). For T2 and T3, statistically significant negative correlations were found for the C5.0 F-measures but positive correlations were found for the SVM MCC measure. Such mixed results also seem to be present in the literature in general. Ho et al. (2006) also use T2 within their research and do not find it to be particularly effective. Due to the similarity of results for T2 and T3, it is suggested that the investigation of T2 should be extended. With dimensionality results, the size of the values is dependent on the size of the datasets. Ho et al. (2006) find in their research that low T2 values generally result in bad performance. Cavalcanti et al. (2012) also find dimensionality to not be particularly useful, however their research only used a Nearest Neighbor classifier. Lorena et al. (2012) on the other hand find T2 and T3 to be highly effective, although the ratios used are slightly different. Clearly, these findings are relevant to this discussion.

When discussing these results, the concept of the *curse of dimensionality* has to be acknowledged. It is specifically relevant as a model with statistical significance is relied upon in this research. The concept of this curse is that when a problem has a large number of dimensions, as is the case in this research, the data quickly becomes sparse (Friedman 1997).

To see how data complexity impacts classifier performance in regard to linearity, three complexity metrics were used. Here, different implications were found for the correlations. For L1, L2, and L3 statistically significant negative correlations were found with the F-measures as expected (see Table 9). Positive significant correlation was found with C5.0 MCC.

Ho et al. (2006) find the linearity measures to be effective in separating complexity spaces. L2 is an especially interesting complexity measure. For instance, Kam Ho and Bernadó-Mansilla (2006) found linear classifiers to perform well with complexity in their nine-dimensional complexity space. Chen et al. (2018) obtain good results in their classification models when the problems were linearly separable and simplicity exists.

Regarding Neighborhood measures, six measures were used. The results for N1, N2, N3 and N4 are consistent, with statistically significant negative correlations for the F-measures.

According to Lorena et al. (2018), the neighborhood measures are the most relevant metrics for a random forest model. Also here, correlation was found between most of these measures and the classifiers. In research by Chen et al. (2018), the authors find that multiple classifiers struggle with complexity in terms of neighborhood, aligning with the dominant findings in this research. Ho et al. (2006) describe N1, N2 and N3 to be very discriminating metrics that had a large impact in their research. As mentioned before, this is also found by Lorena et al. (2018). A high correlation between the three was also identified in the same research, matching the results here. The same authors (Lorena et al. 2018) find that N1 and N2 are sensitive to the so called ’labeling noise’. Further, Ho and Basu (2002) note that high values of these measures can also be obtained for problems that are linearly separable. Cano (2013) critiques the measures N1, N2 and N3, saying that their definition means "that C5.0 and SVM are not able to approximate their models to the original instance distribution". For the 3NN Classifier however, they found a strong relationship.

Many researchers do not include the other neighborhood measures (N4, T1, LSC), limiting the comparative baseline. Interestingly however, Cano (2013) notes that the N4 measure was not found to be important, whereas here, the measure shows similar correlations to N1, N2 and N3. For the measure T1, the same author (Cano 2013) finds that it is not helpful in predicting classifier behavior, but this is not supported by our research. Lorena et al. (2018) on the other hand identify it as one of the complexity metrics that had the most impact. LSC mostly did not provide significant correlations in our research and is rarely discussed in the literature.

The three measures ‘Density’, ‘ClsCoef’ and ‘Hubs score’ were used for the category network. *Density* is negatively and statistically significantly correlated with F-measures. NB MCC shows positive correlation that is statistically significant at the 95% level. For the other two measures, the correlations were largely insignificant.

Lorena et al. (2018) identify density as one of the most relevant metrics to their random forest model. In Cavalcanti et al. (2012) however, the measure does not provide valuable information to a nearest neighbor model and is therefore dropped.

Garcia et al. (2015) find the measures density and hubs are effective in capturing the presence of label noise in classification datasets. In our research however, significant correlations for hubs were not seen.

In research on network metrics in SDP, Prateek et al. (2013) find the hubs to not be an important metric in the PROMISE repository, which is also part of the Unified Bug Dataset. In the same research, the authors find the Clustering Coefficient (ClsCoef) to be an important metric in the PROMISE repository. Within our research, no strong conclusion could be drawn for these two measures.

The four measures used for overlap largely show inconsistent and insignificant results. This is surprising as other research has found it to significantly impact classifiers. For instance, Pascual-Triana et al. (2021) find in their research that class overlap negatively affects the performance of the kNN, decision trees and Naive Bayes classifiers. Cano (2013) finds an affect of F1, F2 and F3 on the classifiers C5.0, 3NN and SVM, although the strongest impact was found for the F1 measure. The measure F4 was added by Lorena et al. (2018) and has not been widely reported on in work on complexity metrics.

Furthermore, the connection between the balance measures and the overlap measures is prominent in the literature. Chen et al. (2018) find the two complexity categories to coexist in many datasets. Moreover, Lorena et al. (2018) and Pascual-Triana et al. (2021) find that large overlap strengthens the effects of imbalance.

Generally, it has been found that class overlap increases the difficulty for the predictors to learn the defective class accurately (Chen et al. 2018), which hinders the performance of all classifiers. Following this, the aforementioned connection between the categories of complexity metrics is logical, as imbalance means that few defective instances exist, and overlap makes it difficult for them to be recognised. In our research, the datasets can be classified as complex in terms of overlap, yet a significant correlation between the overlap measures and classifier performance could not be identified, except for the SVM MCC measure. Therefore, further research is needed to assess the impact of overlap on different classifiers in SDP.

As for why the results are unclear, Hu et al. (2009) identify a nearly normal distribution of classes as an ideal environment for F1. As the distribution of classes with defects is right skewed, with fewer defective classes, the results may have been distorted for this measure (Ferenc et al. 2020). Lorena et al. (2018) additionally find that F2 is highly dependent on the number of features in a dataset. Again, further research is needed using the complexity metrics with feature selection algorithms.
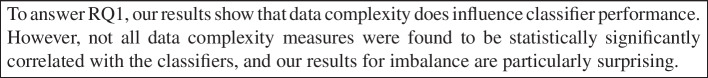

**Table 10:** Domains of Competence

	*C5.0*	*Naive Bayes*	*Artificial Neural Network*	*Random Forest*	*SVM*
*Balance*	Inconclusive	Good	Inconclusive	Inconclusive	Inconclusive
		Behaviour			
*Dimensionality*	Bad Behaviour	Inconclusive	Inconclusive	Inconclusive	Inconclusive
*Linearity*	Inconclusive	Bad Behaviour	Bad Behaviour	Bad Behaviour	Bad Behaviour
*Neighborhood*	Bad Behaviour	Bad Behaviour	Bad Behaviour	Bad Behaviour	Bad Behaviour
*Network*	Bad Behaviour	Inconclusive	Inconclusive	Inconclusive	Inconclusive
*Overlap*	Inconclusive	Inconclusive	Inconclusive	Inconclusive	Bad Behaviour

#### Research Question 2

As previously discussed, the different classifiers performed differently in regards to different data complexity metrics. Consequently, certain domains of competence were indicated for the different classifiers. This is summarised in Table 10 which indicates whether the respective classifier behaves well (‘Good behaviour’) or not (‘Bad behaviour’) when high data complexity exists in each category of the data complexity metrics (Luengo and Herrera 2010). This classification considers overfitting and test accuracy.

As is described in § 3.2, the higher the entropy of class proportions’ (C1) value, the less imbalance and therefore less complexity is evident. Contrary to the interpretation of C1, a higher value of the imbalanced ratio (IMC2) indicates higher imbalance (and higher complexity). Therefore, it can be observed in Table 9 that NB performs well when there is imbalance, and was the only classifier where a conclusive outcome could be reached. Generally however, imbalance is known to reduce classifier performance (Pascual-Triana et al. 2021; Rodriguez et al. 2014). Further, Japkowicz and Stephen (Japkowicz and Stephen 2002) specifically found imbalance to be troublesome for classifiers. As our research contradicts these findings, this requires further research before NB can be recommended for imbalanced problems.

For the three dimensionality measures, a higher value indicates higher complexity (see § 3.2). Therefore, it was found that C5.0 does not perform well when there is data complexity in terms of dimensionality.

For all linearity measures, a higher value indicates higher complexity (see § 3.2). Therefore, the widespread significant negative correlation indicates bad behaviour with increased complexity for most classifiers. The same can be concluded for neighborhood measures, where also higher complexity metric values indicate higher complexity, except for LSC (see § 3.2). This research’s findings regarding linearity and neighborhood aligns with some previous findings (Menzies et al. 2007; Rodriguez et al. 2014). Therefore, the classifiers researched here can not be recommended for problems with high complexity regarding linearity and neighborhood.

Further, C5.0 was found to perform badly with network data complexity and was the only one to provide conclusive results for that complexity metric cluster. Here, higher Density and ClsCoef values indicate higher complexity, and higher Hubs values lower complexity (see § 3.2).

Finally, SVM was found to have bad behavior when overlap complexity exists, as higher values for the metrics in the overlap cluster indicate higher complexity.

To clearly recommend classifiers for problems with certain complexity characteristics, further research should be done. Based on our research alone, NB could be recommended for problems with high imbalance. However, literature has found contrary results. Our results do confirm prior research in regards to complexity in linearity and neighborhood, for which all five classifiers displayed bad behaviour and can therefore not be recommended.
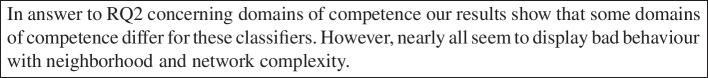


## Threats to Validity and Limitations

Some threats to the validity of this research have to be acknowledged:

- One threat to validity is that as described in the methodology, the datasets had to be pre-processed manually. For this, a unique identifier had to be created by merging two columns so that the right instances could be extracted from the Unified Bug Dataset.
- Other studies have identified interactive effects of different measures. This is not taken into account within this research but may provide further interesting insights that change the interpretation of the results here.

Furthermore, a number of limitations of the research have been identified.

- The scope of the study was limited to the Unified Bug Dataset. Although this is a collection of datasets that each are relatively large in size in the domain of SDP, other datasets may produce different results.
- Comparisons with other studies, models and datasets can only be made when the same software metrics exist and are given, as these are used by the model to predict defects. As was found in the Unified Bug Dataset, different software metrics are given for each dataset.
- The scope of the study was also limited to standalone classifiers. As touched on in the literature review, classifiers combined with certain pre-processing steps such as ensembles can increase the performance of a model and help to deal with complexity in data.

## Conclusions and Future Work

Within the settings of this research, trends were found with the behaviour of machine learning classifiers and the chosen performance measures. This underlines that both ML classifiers and performance metrics are specialised and have their own domains of competence. In particular, it can be concluded that the F-measures behave similarly whereas the MCC measures behaves differently in most cases. Perhaps most surprising are the findings for the balance measures, as higher imbalance was correlated with good classifier performance for the MCC metrics, contradicting many findings in the research. However, all data complexity metrics were found to have some problems for the classifiers, although there are classifiers that excel with some of these challenges. As consistent implications could not be identified for all data complexity categories, our methodology should be explored further. Also, the performance measures should be explored more extensively, as their results were found to vary considerably.

To sum up, our results show that data complexity does influence classifier performance. Our results also show that the classifiers’ domains of competence differ. However, Naive Bayes seems to behave well for Balance.

Our further contribution is the analysis of the Unified Bug Dataset from a perspective of some data complexity metrics. To further encourage future research, datasets, code and methodology have been made publicly available.

Thus, this work contributes to the existing body of knowledge by investigating SDP from the viewpoint of certain data complexity metrics for classifiers. We also suggest that the ECoL library classifiers are under-researched in the present literature.

We encourage replication and further development of our work. There are a number of possible directions for future research.

- Expand the research to more classifiers to find further domains of competence. This should also include classifiers combined with pre-processing steps that specialise in certain complexity areas.
- Replicate the study with different datasets from SDP, to check whether similar results are found or if (for instance) more intuitive results are found for the data complexity metrics such as Balance as discussed previously.
- Cross validation could be performed to further ensure that the splitting of the datasets for training and testing is optimal.
- As this was not done here, the combined effects of the data complexity metrics could also be measured using the model proposed here and some variations thereon.
- Research the ECoL library classifiers more extensively.
- Check the correlations using non-parametric statistical tests.

## Data Availability

Data and scripts are available at: https://github.com/JonasEberlein98/ComplexitySoftwareDefectsDatasets Original datasets are available at: http://www.inf.u-szeged.hu/$\sim$ferenc/papers/UnifiedBugDataSet/
